# Identification and Spread of the Ghost Silverfish (*Ctenolepisma calvum*) among Museums and Homes in Europe

**DOI:** 10.3390/insects13090855

**Published:** 2022-09-19

**Authors:** Pascal Querner, Nikolaus Szucsich, Bill Landsberger, Sven Erlacher, Lukasz Trebicki, Michał Grabowski, Peter Brimblecombe

**Affiliations:** 1 Natural History Museum Vienna, 1. Zoology, Burgring 7, 1010 Vienna, Austria; 2 Institute of Zoology, University of Natural Resources and Life Sciences, Gregor-Mendel-Straße 33, 1180 Vienna, Austria; 3 Natural History Museum Vienna, Central Research Laboratories, Burgring 7, 1010 Vienna, Austria; 4 Rathgen Research Laboratory, Staatliche Museen zu Berlin, Stiftung Preußischer KulturbesitzSchloßstraße 1A, 14059 Berlin, Germany; 5 Museum für Naturkunde, Moritzstraße 20, D-09111 Chemnitz, Germany; 6 Department of Invertebrate Zoology & Hydrobiology, University of Lodz, Banacha 12/16, 90-237 Lodz, Poland; 7 Department of Marine Environment and Engineering, National Sun Yat-Sen University, Kaohsiung 804, Taiwan

**Keywords:** introduced pest, invasive species, DNA barcoding, identification key, monitoring, insect traps, Lepismatidae

## Abstract

**Simple Summary:**

*Ctenolepisma calvum* was first described in Sri Lanka (Ceylon) in 1910. Up until today, it has only been identified within buildings, and its natural habitat is unknown. In 2007, it was discovered in Germany and was considered a neobiotic species of Lepismatidae in Europe. This led us to analyze the available data of the first occurrences in European homes and museums. Our observations indicate that it is possibly distributed with materials (packaging material, hygiene articles, paper, cardboard, and collection items). Little is yet known about the biology of this introduced pest. We describe its preferred habitat within buildings, its climate requirements, and its potential to act as a new museum pest in Central Europe. A simple morphological key can help correct species identification.

**Abstract:**

*Ctenolepisma calvum* was first described in Sri Lanka (Ceylon) in 1910, and this island is probably the origin of this species. Later, it was also found in the Caribbean (Cuba and Trinidad and Tobago). Up until the present, it has only been identified within buildings (a synanthropic species), and its natural habitat is unknown. In 2007, it was discovered in Germany and was considered a neobiotic species of Lepismatidae in Europe. It has rapidly spread throughout Europe and beyond in recent years. This led us to analyze the available data of the first occurrences in Germany, Austria, and other European countries. Furthermore, we compared the spread inside of museums in Vienna (Austria) and Berlin (Germany). These museums have been monitored for a long period with sticky traps, representing the best source of information on the dispersion dynamics of *Ctenolepisma calvum*. We found a scattered occurrence of this species in 18 countries in Europe (including Russia and Ukraine). The first record for Poland has not previously been published; however, this species has been present there since 2014. Surprisingly, it was found in Hungary in 2003, but a record was only published online in 2021. Additionally, in Germany and Austria, where most data are available, the spread of the species does not follow any clear pattern. In museums in Berlin, the species has only been found in one location. In contrast, the species rapidly spread in museums in Vienna between 2014 and 2021, from four to 30 locations, and it is now a well-established species with occasional high abundance. We examined the spread of the species at three spatial scales: (i) Europe, (ii) national, and (iii) regional. Our observations indicate that it is possibly distributed with materials (packaging material, hygiene articles, paper, cardboard, and collection items). Little is yet known about the biology of this introduced pest. We describe its preferred habitat within buildings, its climate requirements, and its potential to act as a new museum pest in Central Europe. This species seems to thrive at room temperature in buildings. Further impact on the species due to climate change in the future is also discussed. We offer a simple morphological key and a detailed identification table to help correct species identification.

## 1. Introduction

Silverfish (Zygentoma) are small arthropods feeding on cellulose-based materials. As an order of primarily wingless insects, their last common ancestor with wings dates back more than 400 million years [1]. Most of the ~600 described species found worldwide live in hot and dry natural habitats; only a small number of Zygentoma are found inside buildings as a synanthropic species. The common silverfish (*Lepisma saccharinum* Linnaeus, 1758) is commonly identified in humid rooms, such as bathrooms and basements, and it may have been associated with humans and the indoor biome for a long time [2].

Recent years have witnessed an increase in synanthropic Zygentoma species in Central Europe, with new species being introduced and spreading across the continent [3,4,5,6,7,8,9,10,11]. At the moment, the six species regularly recorded in Central Europe are the common silverfish *L. saccharinum*, the firebrat *Thermobia domestica* Packard, 1873, *Atelura formicaria* Heyden, 1805 (a species living in ant nests), and three recently introduced species: the invasive gray or long-tailed silverfish *Ctenolepisma longicaudatum* Escherich, 1905, the four-lined silverfish *C. lineatum* (Fabricius, 1775), and *C. calvum* (Ritter, 1910). *A. formicaria* is mainly restricted to habitats outside of buildings [12], while all other species are mostly related to human activity and found interiors. *C. lineatum* is also found outside of buildings in some European countries; it is a facultative synanthropic species. Additionally, *Coletinia maggii* (Grassi, 1887) is mentioned uniquely in Austria [13]. *Nicoletia phytophila* Gervais, 1844, *Stylifera impudica* Escherich, 1905, *Gastrotheus ceylonicus* (Paclt, 1974), and *Ctenolepisma rothschildum* Silvestri, 1907 have been reported at least once in Germany [14], but they are not known as common insects in homes and buildings.

Silverfish are indoor pests in homes, offices, museums, and galleries, causing harm to objects such as paper, books, photographs, and wallpaper [15,16,17,18,19,20,21]. They are, thus, an important target of investigation (monitoring with traps) and integrated pest management [15,18,20]. The insects are rather shy and night-active; thus, they are often overlooked. In museums, *C. longicaudatum* is a recognized pest, where it has damaged a number of objects in recent years. Although such risks are known for *L. saccharinum*, the species seldom causes damage to objects. It requires high humidity (>70% RH); hence, only paper-based material stored in damp rooms and on the ground can be damaged. Aak et al. [3] showed a widening spread and particularly northward movement of the long-tailed silverfish *C. longicaudatum* in Norway. In homes, most silverfish (Zygentoma) are not real pests, but only nuisance animals.

**Figure 1:**
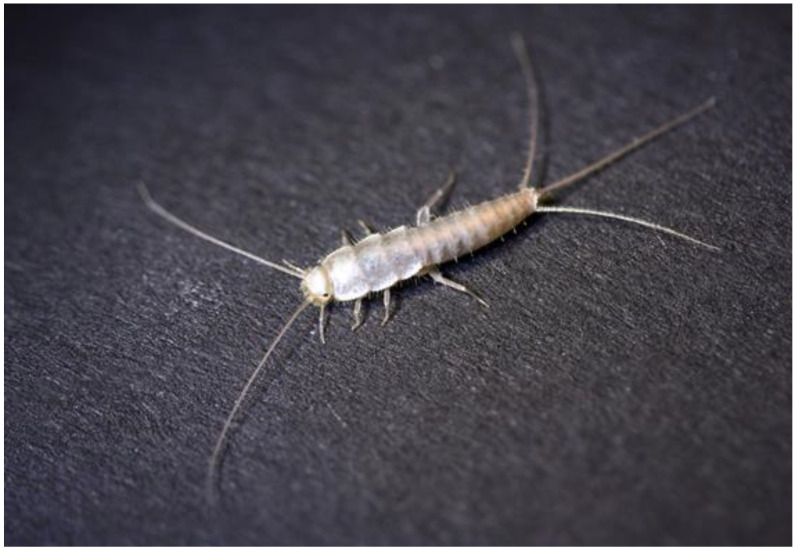
First image of *Ctenolepisma calvum* in Germany (Photograph: Sven Erlacher, Museum of Natural History Chemnitz).

*C. calvum,* described in Sri Lanka (Ceylon) in 1910 [22], was initially discovered in Chemnitz, Saxony almost 100 years later, in 2007 [23], long considered the first occurrence in Europe. In Germany, it is called ‘Geisterfischchen’ (small ghost fish) because of its small size and white pearlescent color; accordingly, we adopted ghost silverfish as the common name (Figure 1). The insect has a fine body; the upper body appears whitish and glossy, with individual upright bristles. Scales all point toward the rear of the body. Cerci are about as long as the body, and they can break off easily with the lightest touch. The insects can grow up to 8 mm in body length (without antenna, cerci, and terminal filum) and regularly shed their skin. Their lifespan and reproductive cycle remain unknown. They are nocturnal. Dry apartments with floor heating, ensuring constant temperatures >20 °C, appear to be a preferred environment ([6] and personal observations of S.E. and P.Q.). *C. calvum* thrives at room temperature and moderate humidity, although the exact climate requirements of the species are still unknown. Since its discovery in Ceylon [22], the preference for higher temperatures and humidity for survival and reproduction is to be expected. Such preferences likewise fit subsequent findings of *C. calvum* in Central America, where it was mentioned as the most common species in human habitation, especially in Havana, Cuba in the 1970s [24,25,26]. Whether it is still widespread in Cuba today remains unknown, as with its occurrence and distribution in homes in Sri Lanka.

In recent years, *C. calvum* has been recorded in different countries of Central Europe [3,6,27,28,29,30,31,32]. The Integrated Pest Management (IPM) textbook by David Pinniger [6] does not include it as a pest species (although *C. calvum* was added in the German translation [33]). The distribution of *C. calvum* was reviewed by Kulma et al. [6] in 2022; however, since the greater part of the observations of this species stems from records during monitoring programs of pest species, our knowledge on the history of the spread of *C. calvum* is still rudimentary, with little known about the biology and ecological requirements. However, it probably occupies similar habitats to those of *C. longicaudatum* (see Lindsey, 1940 [34] for this species). *C. calvum* has spread rapidly in Germany and Austria. Neither species (*C. calvum* and *C. longicaudatum*) can survive a winter outside with freezing temperatures; thus, it is probable that they are transported on materials from one building, city, or country to another.

*C. calvum* and other species of Zygentoma, e.g., *L. saccharinum* and the invasive *C. longicaudatum*, are regularly found in museums on insect traps. Correct identification of the species aids in understanding the introduction of a new pest species to a museum, as well as the spread within a city, country, or continent. We combined records of *C. calvum* in biological databases and national species lists with the monitoring data from museums. Rapid change makes the widening presence of *C. calvum* worthy of study. We investigated the changing geographical range and the increase in catch (locations and abundance) from 2007 onward. The study provides an opportunity to assess likely changes in the future and give a sense of issues that might require more consideration in pest management protocols. 

This paper aims to update the distribution area and spread of this neobiotic species in Europe. We look at three spatial scales: (i) Europe, (ii) national (Germany and Austria), and (iii) regional (Berlin and Vienna). The patterns observed allow us to explore the spread of these insects through Europe and requirements for survival at new locations.

## 2. Material and Methods

### 2.1. Data Sources

We analyzed records from a number of academic sources (papers, articles in local languages, and gray literature) and a range of databases to gain insight into the spread of the neobiotic species, *C. calvum*:

(i) Sven Erlacher (coauthor of this paper) found the species in Europe in 2007 and first revealed it in 2017 [23], before seeking further findings via the internet. Records from many locations in Germany and other European countries were, thus, obtained by him showing the early occurrence and distribution of the species in Europe, with identification based mostly on photographs.
(ii) Dataset based on iNaturalist (https://www.inaturalist.org, accessed on 31 March 2022) [35], where pictures of animals are posted and identified with the help of experts. Many examples of *C. calvum* and other Zygentoma species in Europe were identified by Nikolaus Szucsich (coauthor of this paper). Identification of pictures has its limitations, especially if scales or other characteristics are lost; hence, confusion of *C. calvum* with some other synanthropic *Ctenolepisma* species is possible. However, few other species in Europe have this white appearance. This database relies very much on the activity of interested naturalists in observing new species and posting them online, along with the experts who identify them. It has a bias that arises from the varying activity among voluntary naturalists in different countries. It also depends on the use of internet resources for insect identification, but is nevertheless effective at indicating the occurrence of new species. We used two online databases: (a) iNaturalist and (b) the Global Biodiversity Information Facility GBIF at https://www.gbif.org, accessed on 31 March 2022 [36]) as reliable aggregators of occurrence. Most observations in GBIF are based on iNaturalist data (identification based on pictures only). Additionally, we sought information from (a) experts on Lepismatidae and Apterygota, (b) museum IPM professionals, and (c) entomologists working in natural history museums across Europe, for recent observations.
(iii) Datasets resulting from the monitoring of insect activity inside museums in Austria, including many locations in Vienna, Berlin (Germany), Switzerland, and Liechtenstein (see Appendix A). These arise as part of IPM programs related to museum pests. Because pests are an important threat to collections, insect activity is monitored very closely using numerous traps in the rooms of many buildings [37,38,39,40,41]. Traps are checked at regular intervals and replaced when needed. Both sticky blunder and pheromone traps have long been found effective in monitoring insects in museums, and they can trap silverfish when placed on the ground (personal observations of authors). The routine nature of these programs means that these data are quite reliable regarding the presence/absence of a species over the course of years. Most sites are in Vienna, followed by other regions in Austria, Berlin, a few locations in Switzerland, and one in Liechtenstein. The number of locations has increased over time as more museums are monitored. In 2021, a total of 31 sites were available in Austria, along with 27 sites in Berlin, six in Switzerland, and one in Liechtenstein.

**Figure 2:**
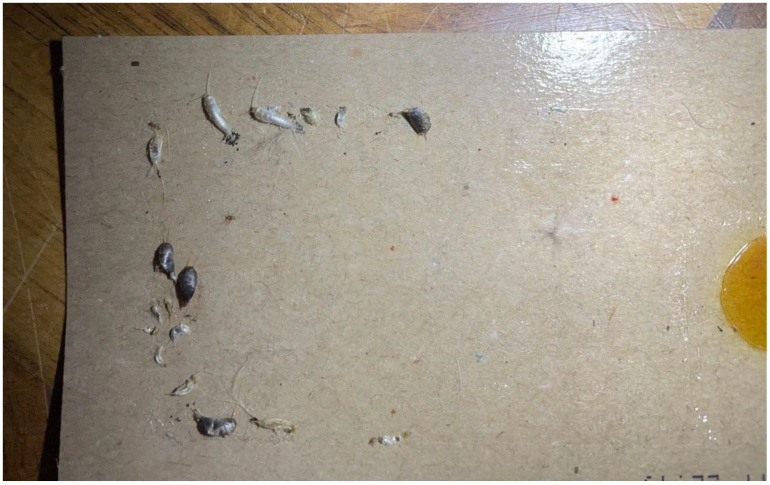
*Ctenolepisma calvum,* together with *Lepisma saccharinum,* on a sticky trap in a museum in Vienna, Austria (Photograph: Pascal Querner, Museum of Natural History Vienna).

Our data cover periods up to 7 years through to 2021 (for one location, at the Kindermuseum in Vienna, the record runs until July 2022). The traps were renewed in the early parts of the year (March) and checked at approximately 2 month intervals across the summer (until October). Figure 2 shows a trap in a museum with some Lepismatidae. Museum collections used the insect blunder trap *Catchmaster* and pheromone trap *Finicon* for webbing clothes moths (*Tineola bisselliella*) in museums in Austria, Switzerland, and Liechtenstein (provided by PPS www.pps-vertrieb.de, accessed on 1 July 2022). In the museums in Berlin, insect blunder traps by Historyonics and the *Finicon* pheromone traps were used (provided by www.historyonics.com, accessed on 1 July 2022). Silverfish seem to enter the clothes moth pheromone traps on the floor, probably because they provide shelter during the day.

Microclimate data were collected using a MostraLog data-logger (www.mostralog.com, accessed on 1 July 2022) placed on the floor next to a trap that had caught high numbers of *C. calvum* over recent years (Kindermuseum in Vienna). Temperature and relative humidity were measured every 15 min from July 2021 until July 2022. This was expected to give an insight into the preferred microclimate of this species in a museum environment, assuming that the insects live, feed, and reproduce close to the trap.

### 2.2. Identifcation

The correct identification is crucial for the discovery of new species in a country. Many new records are based mostly on photographs. We believe that, if the species is found indoors and it shows the typical morphological characters, this is a very good indication, that it is *C. calvum*. Nevertheless, for each new record, further morphological characteristics (combs for example), beyond the color, eyes, pigmentation, and body size, should be confirmed (see Supplementary Tables S3 and S4 for identification key and table with all characteristics). Good pictures of these characteristics can be found in [6].

### 2.3. DNA Barcoding

The DNA barcode sequences provided for this study were obtained from three individuals of *Ctenolepisma calvum* and, for comparison, from eight individuals of *Lepisma saccharinum* collected in Lodz, Poland (Supplement Table S1). Total genomic DNA was extracted from a leg of each specimen, using the Chelex-based method [42]. The COI gene fragment was PCR-amplified with the primer pairs LCO1490-JJ/HCO2198-JJ [43]. The reaction conditions followed Hou et al. [44], modified by Rewicz et al. [45]. The PCR program was as follows: initial denaturing for 60 s at 94 °C, followed by five cycles of 30 s at 94 °C, 90 s at 45 °C, 60 s at 72 °C, then 35 cycles of 30 s at 94 °C, 90 s at 51 °C, 60 s at 72 °C, with a final 5 min extension at 72 °C. After amplification, 2 μL of each PCR reaction was analyzed by electrophoresis on 1% agarose gel to verify the product length and quality. Prior to sequencing, 5 μL of PCR products containing visible bands were purified with Exonuclease I (2 U, EURx) and alkaline phosphatase Fast Polar-BAP (1 U, EURx), according to the manufacturer’s instructions (Merck KGaA, Darmstadt, Germany). Sequencing of the purified PCR products with forward primer was outsourced to Macrogen Europe BV. The sequences were aligned in Geneious 10.2.6 (Biomatters Ltd., Auckland, New Zealand) software package [46], using MUSCLE [47] with default parameters. The resulting alignment was edited and trimmed of primers, and then checked for the absence of stop codons, double peaks, and frameshifts using Geneious 10.2.6 (Biomatters Ltd.). All DNA barcode sequences generated for this work were deposited in BOLD Systems (https://www.boldsystems.org/, accessed on 1 July 2022) and GenBank (https://www.ncbi.nlm.nih.gov/, accessed on 1 July 2022) as in Table S1, along with relevant voucher information, taxonomic identification, and photos. The molecular data are publicly accessible through the dataset DS-PLLEP (dx.doi.org/10.5883/DS-PLLEP), in BOLD (www.boldsystems.org, accessed on 1 July 2022) [48], and in GenBank (accession numbers in Supplement Table S1). The neighbor-joining distance tree [49] was constructed on the basis of the K2P distance [50], with bootstrap values calculated using 500 replicates [51] as implemented in MEGA X [52]. The publicly available COI sequences of *C. calvum* identified only as Zygentoma (BIN BOLD:ACA5333) and *C. longicaudatum* (BIN BOLD:AEE2038) were obtained from BOLD, and dataset DS-PLLEP (dx.doi.org/10.5883/DS-PLLEP) was included for comparison.

### 2.4. Statistics

Insect trapping data are necessarily integers and traps frequently have no silverfish at all; therefore, care over the nonparametric nature of the values becomes important in analyzing the catch. We often present central tendency as a median and dispersion in terms of lower and upper quartiles (i.e., *Q*_1_ and *Q*_3_). Such data are sometimes represented as box-and-whisker plots. Catch rate (total catch divided by the number of traps) was used, although we are aware that this can be biased toward smaller catch rates in museums with a large number of traps. The Mann–Whitney test was used as a nonparametric equivalent of the *t*-test, and the Kendall *τ* test was used in place of the usual Pearson regression coefficient. 

## 3. Results

### 3.1. First Discovery and European Scale

The datasets of Sven Erlacher and the online records from GBIF and iNaturalist show the first descriptions from Germany in 2007, albeit published online only in 2017. Between 2007 and 2017 *C. calvum* was found in different regions of Germany, and then in Austria (2010), Teneriffe (2012), Finland (2017), and Switzerland (2017) (see also the analysis of [6] derived from GBIF and iNaturalist [35,36]). Our search for new records in Europe found *C. calvum* in Sátoraljaújhely, Hungary (published online 2021 in Hungarian [53]); it was found in Hungary as early as 2003, and again in 2006 and 2021. This identification by the biologist Gábor Hegyessy of the Kazinczy Ferenczy Museum in Budapest seems trustworthy, making it the first record in Europe, predating the earliest German record by 4 years. Picture of the animals from Hungary were sent to the authors for this paper for confirmation. There is an unpublished record for Poland from 2014 (Appendix B) and a new record in Kraków for 2022 [54]. In 2018, a new record derived from Norway [3], whereas 2020 reveals the insect in Italy and Russia, and one was observed in Slovakia in 2022. The record from Portugal from Vila Real de Santo António is close to the border with Spain, just across the Guadiana River. The largest number of new records emerged in 2021, where the species was recorded in eight new countries (Luxembourg, Croatia, Czech Republic, Spain, Kosovo, Slovenia, Ukraine, and Portugal). The GBIF and INaturalist databases list a number of isolated occurrences of *C. calvum* outside Europe: Venezuela (2017), Mexico (2020), Singapore (2020), Canada (2021), Vietnam (2022), and Japan [35,36] (unpublished data for Japan). There have been no detections in the UK, France, the Netherlands, Belgium, Liechtenstein, or Portugal (local experts contacted are listed in Supplementary Table S2). 

**Figure 3:**
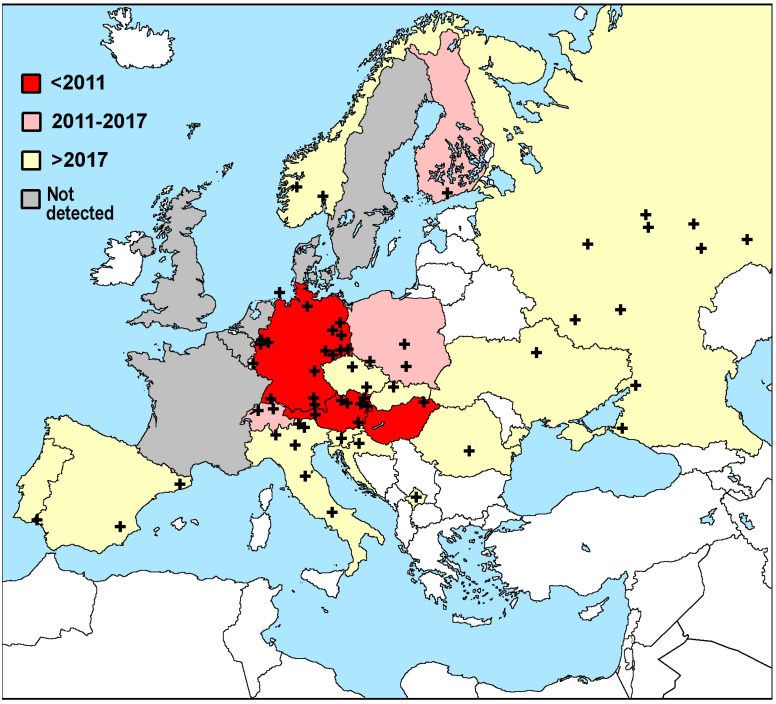
Map of the occurrence of *Ctenolepisma calvum* in Europe. The year of the first observation in countries is denoted by the shading. Places within countries where the insect was initially found are marked as +. Gray shading denotes countries where traps and observations have not revealed *C. calvum* (details in Supplement Table S2). White indicates countries, where the species has not yet been found (because of lack of traps, experts, or introduction).

Reports of *C. calvum* in Europe are mapped in Figure 3 and Supplement Table S2, which give hints of a widening distribution, although care is needed as it could merely reflect a widening interest in *C. calvum*, but the later discussion of its absence may give a better view of the extent to which the distribution has truly widened. The first occurrence recorded in individual countries is denoted by different colors, while sites where *C. calvum* has been found are marked as crosses on the map. This does not capture multiple catches or observations at nearby sites, although the full dataset is available on request. 

### 3.2. National Scale: Germany

Reports from museums and homes across Germany start with an observation from Chemnitz in Saxony as early as 2007, then in Potsdam and Darmstadt (both 2016), and more widely across Germany in 2017. Since 2016, it has also been found in the “Museumsetage im Tietz”. By 2018, *C. calvum* was found as far north as the East Frisian islands (Spiekeroog) and in the south in Munich. New reports suggest that it continues to be recorded in some cities, added by Sven Erlacher to his national database (the most recent records are not indicated in our map, which shows only the first observation in a location).

### 3.3. Regional Scale: Berlin

In Berlin, *C. calvum* was discovered prior to 2017 (two records in the database of Sven Erlacher), but it is still very rare in museums. Out of 27 locations with an intensive insect monitoring program, including large museums (e.g., Bode Museum, Ethnological Museum, National Archive, and two large libraries), it was only found at a single location in 2017, the Archäologisches Zentrum in Berlin (52.52° N, 13.39° E), a newly constructed building from 2012. The findings illustrate a slow increase in catch between the years 2017 and 2021: in 2017, one specimen; in 2019, three specimens; in 2020, six specimens; in 2021, as many as 44 specimens. When only small numbers are found, it is not clear if a self-sustaining population has been established or reintroduction is occurring. However, larger numbers suggest a population that reproduces on site. The monitoring data from the museums of Berlin are based on thousands of traps across these 27 locations. In the Archäologisches Zentrum alone, 204 sticky blunder traps (for all pests) and 55 live traps (primarily for the *C. longicaudatum*) are checked and replaced regularly. Nevertheless, *C. calvum* can be considered as very rare in the museums of Berlin, with the Archäologisches Zentrum is the only site with an established population. This is somewhat different from the situation in Austria, which shows notable increases in Vienna, as discussed below. 

### 3.4. National Scale: Austria

**Figure 4:**
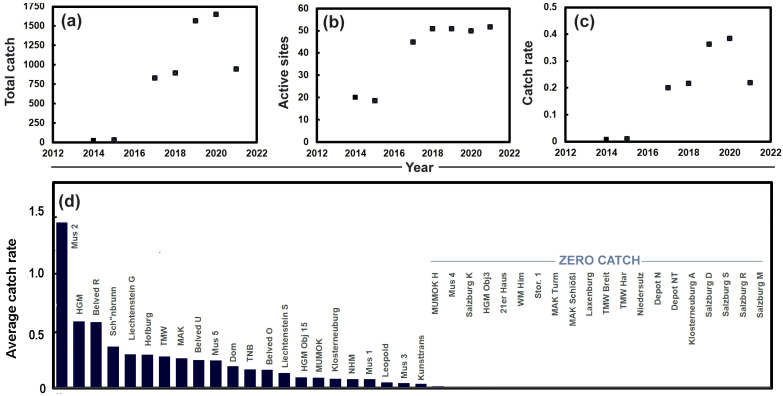
Catch of *Ctenolepisma calvum* in Austrian museums: (**a**) total annual catch; (**b**) percentage of Austrian museums finding *C. calvum*; (**c**) catch rate in Viennese museums; (**d**) average catch rate in Austrian museums (i.e., sum of catches in 2014, 2015, 2017–2021 divided by number of traps deployed at each site). Note: abbreviations are listed in Supplement Table S1.

*C. calvum* was recorded for the first time in 2010 in Vienna, Austria, and in 2012 in Steiermark, as noted in Sven Erlacher’s database. In 2014, the silverfish was found for the first time in museums in Vienna. Since then, its abundance and presence have continued to increase. In the years 2014 and 2015, it was found in four out of 21 museums sampled across Austria. The number of sites increased over time, and, in 2019, it was found in 29 locations (out of 52); in 2021, it increased again to 30 locations across Austria (out of 53 sites), with only spring/early summer catches available for 2022. Insect traps from Austrian museums provide a clear picture of an increasing catch of *C. calvum* (Figure 4a), as well as a sharp rise in the number of museums where the insect is present (Figure 4b). These increases are predominantly observed in Viennese museums, where the catch rate has risen sharply (Figure 4c). In some locations, high numbers, up to 250 individuals, are caught every year. The cumulative 7 year catch rate (i.e., sum of catches in 2014, 2015, 2017–2021 divided by the number of traps deployed at each site) shows that museums outside of Vienna (especially Salzburg) have remained largely free of *C. calvum* (Figure 4d).

### 3.5. Regional Scale: Vienna

**Figure 5:**
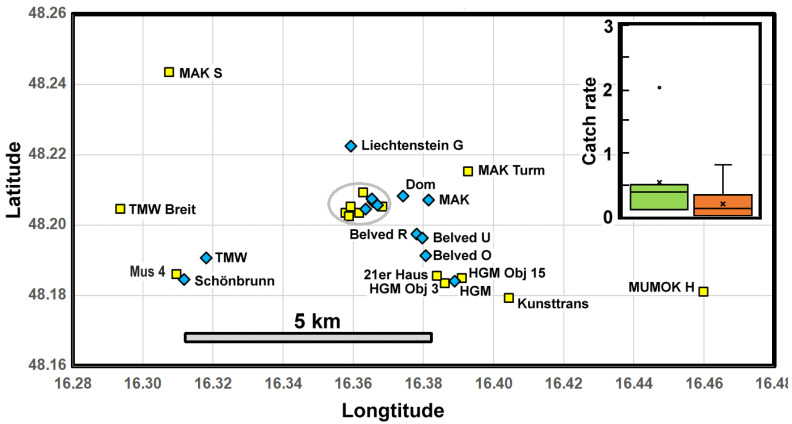
Coordinates of Viennese museums with high catch rates >0.02 for *Ctenolepisma calvum* (blue diamonds) and low catch rates (yellow squares) over the 5 years 2017–2021. Note: The gray ellipse encloses Hofburg, Leopold, Liechtenstein S, Mus 1–3, Mus 5, MUMOK, and NHM, with abbreviations listed in Supplement Table S1 Inset: Box-and-whisker plots of catch rates at the popular museums (green) and less visited sites (red). Note: The quartiles *Q*_1_ and *Q*_3_ define the box, the horizontal line defines the median, the cross defines the mean, whiskers define points within 1.5 times the interquartile range, and isolated points are outliers.

The locations of trapping sites in Vienna are shown in terms of their geographical coordinates (Figure 5). The museums with high and low average catch rates for the period 2017–2021, marked in orange, show little geographical coherence (Figure 5). However, the inset suggests that the popular museums have higher catch rates, hinting that these might be potentially more at risk of importing *C. calvum*. The median catch rate at the popular museums (pm), i.e., Belvedere O., Hofburg, Mus 1, Mus 2, NHM, Schönbrunn, and TMW, is 0.38 (*Q*_1_ = 0.16 and *Q*_3_ = 0.45), while, at the smaller sites (ss), the median is 0.13 (*Q*_1_ = 0.01 and *Q*_3_ = 0.33), a difference marginally significant according to the Mann–Whitney test (*n*_pm_ = 7, *n*_ss_ = 21; *p*_2_ = 0.06).

### 3.6. Other Museums across Europe

Few museums were sampled in Switzerland, but the insect was also recorded there in 2019, with two individuals (at one location out of the three). In 2020, *C. calvum* was found in three museums (out of six in total), while it was found in two in 2021. The catch is very low with only two insects being caught in 2019, four in 2020, and 11 in 2021. At two locations in 2021, the numbers seemed to increase. In Liechtenstein, one location was sampled with traps, but *C. calvum* was not found. Furthermore, a local biologist contacted said that the species has yet to be found.

For many other countries and museums across Europe, we contacted local experts and asked about observations of this new species. It was reported to be absent from the UK, Sweden, France, Denmark, Belgium, and the Netherlands. We believe that negative records are a relatively trustworthy resource, as they are based on good data (thousands of traps, e.g., in the UK, and well-educated and thoughtful IPM coordinators). In some countries in Europe, monitoring in museums is not so widespread; here, the species can more easily be overlooked.

### 3.7. Microclimate of C. Calvum

**Figure 6:**
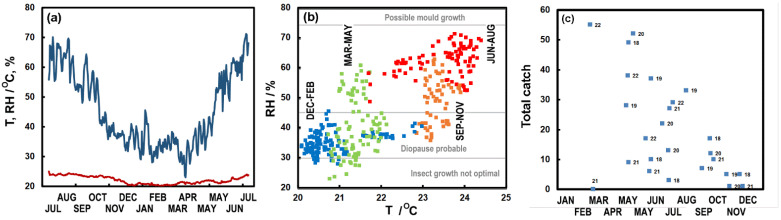
(**a**) Trend in temperature and relative humidity in the Kindermuseum Schönbrunn between July 2021 and July 2022. (**b**) The relationship between daily average temperature and relative humidity in the Kindermuseum for various seasons. (**c**) Catch of *Ctenolepisma calvum* on the traps at various points throughout the year (small numbers 18–22 denote the years 2018–2022 associated with the catch). Note: Growth ranges in (**b**) were taken from [57].

As of yet, there are no measurements from museums to define a suitable microclimate for this species. Microclimate monitoring from the Kindermuseum, Schönbrunn, where there is a high catch rate (Figure 6), derives from a MostraLog data logger (part of a larger project on climate and museum pests [55,56]). This shows stable and warm temperatures (~20 °C) in the museum. The RH fluctuates between 35% and 65% over the year, but does not go above 70%. It seems that *C. calvum* can thrive in these climates, with as many as 55 being caught in late February 2022. Figure 6c shows the declining catch through the year (Kendall *τ* = −0.38, *p*_2_ = 0.008). However, the seasonality of the catch may seem unexpected as most *C. calvum* are caught in early spring when conditions are cool. However, the museum is at a reasonable temperature throughout the year, which may not limit insect activity.

### 3.8. DNA Barcoding

**Figure 7:**
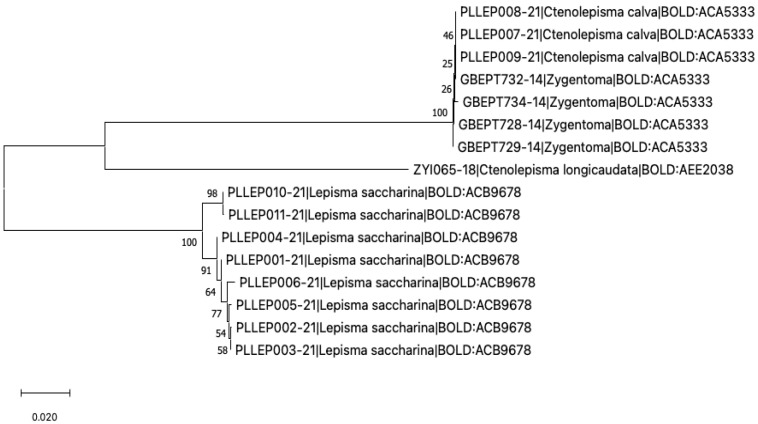
Neighbor-joining tree of the COI sequences representing *Ctenolepisma calvum*, *C. longicaudatum*, and *Lepisma saccharinum*. Numbers next to nodes are bootstrap values.

The final alignment of 16 COI barcoding sequences comprised 566 base pairs. The nucleotide sequences could be translated into amino-acid sequences without any stop codons; the transition-to-transversion ratio amounted to 1.33. According to the neighbor-joining distance tree, the sequences were clustered into well-supported clades (Figure 7), each grouping members of *C. calvum*, *C. longicaudatum*, and *L. saccharinum* respectively. Among these, *C. calvum* was represented by two haplotypes (one of them in Poland, two in Germany), differing only by one substitution.

## 4. Discussion

The rapid spread of this neobiotic species is evident across Europe in recent years. As the insects cannot survive a winter in most Central and Northern European climates, they are distributed by human activity only. They seem to find suitable living conditions within homes, museums, and other buildings. It may be assumed that the species is still widening its distribution, but occurs only within buildings; as of yet, it has not been found outside. 

### 4.1. Distribution in Europe

Currently, the species has been reported from 18 countries in Europe (including Tenerife (Spain), Russia, and Ukraine), and about eight more worldwide (Tenerife was not counted as a separate country as it belongs to Spain). Since its description in Sri Lanka in 1910, it has not been reported on a regular basis; however, this might be related to the lack of interest in or false identification of this small species. It was probably recorded again on the island during a PhD study on IPM in the national library in Sri Lanka in 2021 [58,59,60]. It was also found in the Caribbean in 1964 [25] (Guyana and Cuba). The collector F. de Zayas from the American Museum of Natural History stated that, considering the Cuban specimens, they were the “common household silverfish” in La Habana. In the 1960s, it was reported as a common pest in homes in Cuba, but no publications about this species can be found in recent years.

The earliest observations in Europe came from Hungary (2003), then Germany (2007), followed by Austria (2010), Tenerife (2012), and Poland (2014). This does not show a clear pattern with a spread from one country to another, although Hungary, Germany, Austria, and Poland are geographically close. This species is spreading mostly in material transportation, not outside of buildings. Therefore, travel over large areas within a few days is possible. However, Finland and Switzerland in 2017 and Norway in 2018 are difficult to explain. In 2020, the ghost silverfish was reported in the Russian Federation (near Kazan, Yekaterinburg, Voronezh, and Taldom, some 100 km north of Moscow) and Northern Italy (Southern Tyrol, Vicenza, and Milan). In 2021, *C. calvum* was also reported in Slovakia, the Czech Republic [6], Kosovo, and the west of Europe, close to the Portuguese–Spanish border, near the banks of the Guadiana River. We have to take care in distinguishing between a single record or observation of a species with respect to that of a resident and reproducing population. Only if insects are repeatedly found in the same location can we assume that a population is thriving at the site. From Norway, many samples were submitted to the Norwegian Institute of Public Health during 2000–2019, and this is one of the best datasets to establish where and when the species was first discovered and the time when numbers increased [3].

The map of Europe (Figure 3) does not suggest a clear north–south or east–west distribution pattern. *C. calvum* seems to have first appeared in Central Europe, i.e., Hungary, and a few years later in Germany. It is not known how or exactly when the insects entered Europe. Nevertheless, the genetic uniformity of samples from Poland and Germany, as shown from DNA barcoding, suggests that they may have come from a single source population. Similarly to *C. longicaudatum*, *C. calvum* is likely transported with packaging material, but their origin remains unknown. Today, it is still primarily found in Central Europe, but is spreading from one country to another. It remains absent or has not been reported in France, the United Kingdom, Ireland, Denmark, Belgium, the Netherlands, and probably large parts of Spain and Portugal. Whether climate is the main factor for limited occurrence is not yet known. There are additionally some Eastern European countries where it has yet to be reported; despite being found in Russia and Ukraine, there is a large gap in between where the silverfish has not been found. 

In Germany and Austria, it has spread within a reasonably short time (2007 until today in Germany) and (2010 in Austria). Our data show that it seems more abundant across Austrian museums compared to those of Germany, as it is only found in one museum in Berlin, compared to 30 in Vienna. However, it was also found in one museum in Chemnitz in 2016 [23] and in recent years in other locations across Germany (data not presented). The ghost silverfish catch generally rose in the most recent years, as well as during the period of COVID-19 enforced closures of 2020, probably as a result of insect ranges within museum buildings increasing because of low levels of human disturbance [27,28]. Popular museums with numerous visitors have higher catch rates, hinting that these might be potentially more at risk of importing *C. calvum*. However, this was not true for all locations in Vienna (some small museums had many individuals), and it was also not found in some large museums in Berlin with many visitors. Whether *C. calvum* is spreading from one museum to another via object loans or mainly other materials is still unknown.

### 4.2. Potential Routes for Dispersion

We now look at the data and explore it at three spatial scales: (i) Europe, (ii) national—in Austria and Germany, and (ii) regional—Vienna and Berlin. The patterns observed do not show clearly how the insects are distributed in Europe at all spatial scales. If they mainly move with packaging materials, the distances between countries, cities, and individual museums or other buildings may not be that relevant, as materials are constantly transported across Europe.

Insect pests (and other arthropods) outside of buildings might actively spread by walking or flying from one location to another, but their speed of dispersal is limited. Urban and indoor environments are often different and more affected by transport of goods and materials into museums [61], and this phenomenon is increasing, as the movement of objects and persons is also increasing [62,63,64,65,66]. It seems likely that the rapid spread through Germany and Austria over the last few years, though poorly understood, may have come as a result of the transport of paper and cardboard packaging materials. 

*C. longicaudatum*, also a neobiotic species, has spread across Europe in recent years. We know that it is transported with packaging materials, museum transport boxes, archive materials, and humans. *C. longicaudatum* is now present in most countries of Europe and in museums, archives, and libraries, where it is a well-known pest with reports of damage to objects. Assuming that *C. calvum* disperses in similar ways to *C. longicaudatum*, it may be that *C. longicaudatum* is a few years ahead of its smaller relative, which is also less obvious, potentially being mistaken for juvenile *C. longicaudatum*.

### 4.3. Ecological Requirements/Limitations

Little is known about the biology and response of *C. calvum* to environmental conditions. However, the widening distribution and increasing presence in museums indicate that it has come to be a significant part of the total silverfish catch in Austrian museums [27,28]. After the first occurrence of the species in Austrian museums, the number of individuals trapped increased, as did the number of locations where it was found. We have to take care with these data as the number of locations where monitoring was installed also increased over the years. In some locations, we can see a large number of individuals in a small space; this could be related to individual traps or rooms, not necessarily a museum exhibition room or storage depository. Sometimes, these insects are found at regular intervals on the traps in entrance halls, kitchens, and reception areas, as well as in archives, historic museum rooms, or storage depositories (e.g., Feuerwehr Museum Vienna, Kindermuseum Schönbrunn, Welt M.). However, across all locations, no objects appear to have been damaged by *C. calvum*, with other pest species being more problematic (e.g., *C. longicaudatum*). After the introduction of new animals into a location, we presume that the microclimate, food availability, lack of competition with other silverfish species, and undisturbed areas are key factors for a stable or increasing population to develop.

### 4.4. Observations about the Biology of the Insects

**Figure 8:**
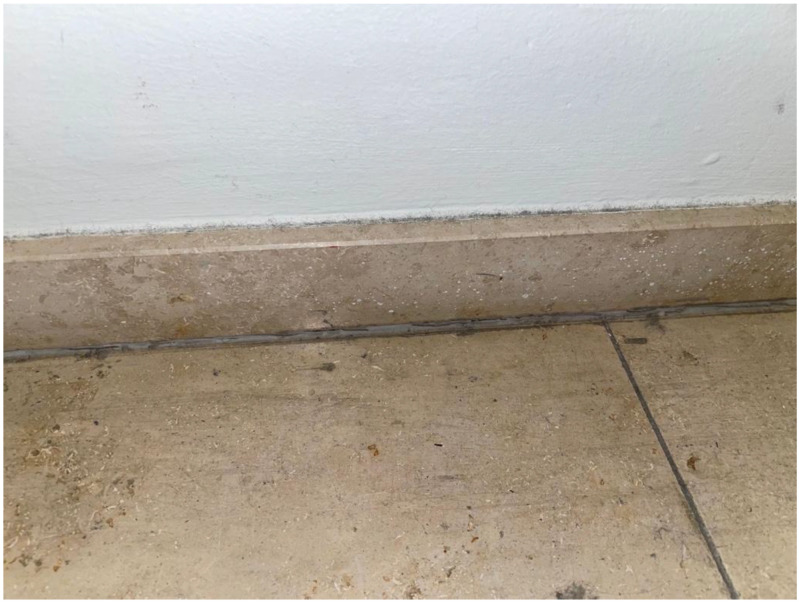
Microhabitat 1 of *Ctenolepisma calvum* in the Firebrigade museum in Vienna with a stone floor.

**Figure 9:**
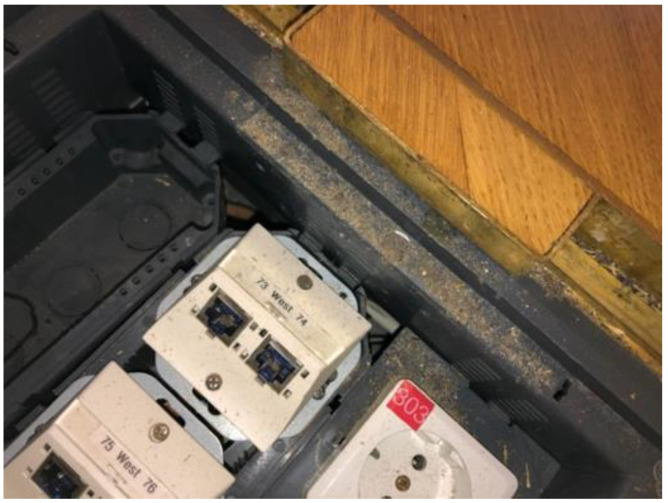
Microhabitat 2 of *Ctenolepisma calvum* (in the center of the photograph) in an electric socket in a museum in Vienna as part of a wooden parquet floor.

Microhabitats, where the insects were observed or trapped, suggest that they like warm and relatively dry climates. They are found in homes with floor heating (personal observations P.Q.), as well as museums with warm and dry interiors. They are also found in rooms with stone (Figure 8) or parquet floors, and they can even hide in electric sockets (Figure 9). When placing a wet textile blanket overnight, with a gap underneath on the floor, live animals are often found beneath in the morning (personal observation S.E.).

As with *C. longicaudatum*, *C. calvum* can climb walls and also hide during the day behind paintings. Microclimate monitoring in locations with a high catch rate in one Austrian museum also confirmed such results (Figure 8 and Figure 9). *C. longicaudatum* needs high humidity for the development of the eggs and nymphs [67]; however, whether this is also the case for *C. calvum* is unknown. It probably has a diet similar to other silverfish species: a combination of cellulose, protein, and probably organic-rich dust and small microorganisms. The life cycle period, from egg stage to adulthood, how often it molts, and how long adults live are still unknown and a matter for future research. Live animals, after collection, can survive for many months in a room climate (22 °C and 55% RH) or a climate favorable for rearing the *C. longicaudatum* or *L. saccharinum* (22 °C and +70% RH), but they do not reproduce and subsequently die. We did not manage to breed the species in captivity; hence, we are unsure of key climate, diet, or substrate factors.

### 4.5. Ctenolepisma calvum as a Pest

So far, there are no reports of damage caused by the species in museums, even where there are large infestations [3,6,7,8,10,11]. In a few cases in Austria, the abundance of the insects was so high that they can be considered a pest or at least a real nuisance animal. A few of those locations with very high numbers and catch rates (see Figure 4) were rooms in museums, where the animals are potential pests to paper-based objects and materials. Compared to the *C. longicaudatum*, no damage to paper has been observed in a collection, archive, or library. The application of ADVION bait gel for cockroaches was tested against *C. calvum* and showed good results (similar to the treatment of the *C. longicaudatum* [67,68,69,70], unpublished data). If objects are infested, they might be treated with humidity, regulated heat, freezing, or anoxia [71]. Furthermore, in one home in Tirol (Austria), many animals were observed in an apartment, which recently required treatment (June 2022). The silverfish are regularly found in the kitchen, in cups and plates, under storage boxes, under other objects and carpets in the living room, and in children’s beds.

### 4.6. C. calvum and Climate Change

Silverfish are rather shy and nocturnal insects; hence, their range within museums increased during COVID-19 lockdowns, when museums were often closed [27,28]. This gives a hint of how the changes in activity and indoor climate can also affect small pests such as silverfish. Climate change that brings increasing warmth is likely to alter insect populations and is particularly relevant to museums [55,72,73,74], and it may be responsible for some increases in population. In the case of Zygentoma [3], they show a widening spread, particularly the northward movement of *C. longicaudatum.* However, urban and indoor environments are often different, and they are also affected by climate control (HVAC systems). 

In line with other insects, such as the webbing clothes moth, silverfish catch in museums has generally been seen to increase [28]. This includes the common silverfish (*L. saccharinum*), as well as the invasive *C. longicaudatum*, increasingly abundant across Europe. High numbers are often present in rooms or on traps, even where humidity is lower than that preferred by the common silverfish. Nymphs need a high humidity, but the larger insects and adults can wander long distances to access new areas with favorable microclimates. The species *C. longicaudatum* has become the most published and talked about museum, archive, and library pest in the last 5–7 years at IPM and urban pest conferences [75,76,77,78,79,80,81] (see [82,83,84,85,86,87,88,89,90,91,92,93,94,95] for its distribution in Europe).

More detailed analyses from Austria benefit from the catch observed in blunder and pheromone traps set out at a range of ground floor locations in the Technisches Museum Wien, Schönbrunn Palace, Hofburg Museum (Silberkammer), and Mus 5. It would further be useful to expand the range of study sites, including those of Sven Erlacher, with traps. 

### 4.7. C. calvum Identification

The correct identification of this species is crucial if we want to know its exact distribution in Europe. Until now, it has only been included in the identification key of Wygodzinsky in 1972 [25]. It is still not found in national identification keys or museum IPM literature [15,18,56,96,97,98] (see the detailed description in [6] with detailed photos of morphological characteristics). We, therefore, present a simple identification key that can be used by a museum conservator or a pest contractor (Supplement Table S3). This key is based mainly on morphological criteria, which can be seen with a good microscope. If *C. calvum* is found, we advise checking morphological details e.g., number of styli or combs on urotergites and urosternites, with the detailed table of the species in homes in Europe (Supplement Table S4). We hope that clear and accurate identification will help to find new locations of this species and gain better insight into the way it is spreading. Identification of pictures was used for many of the first records across Europe, but only if we were confident with the quality of the photographs. This is a limitation of this study; therefore, confusion of *C. calvum* with some other synanthropic *Ctenolepisma* species is possible. The addition of DNA barcoding will help in future correct identification.

## 5. Conclusions

Recent years have witnessed an increase in synanthropic Zygentoma species in Central Europe, with new species being introduced and spreading across the continent. However, relatively little is known about the silverfish species *C. calvum*, but it has spread within a rather short time through Europe since its first discovery in Hungary 2003 to many other countries. It is not clear how it was introduced into Europe, and this will probably remain a mystery. As with the relative species *C. longicaudatum*, *C. calvum* is closely associated with human activities, andm in many regions in Europe, it cannot survive a winter outside. This combination tells us that it must be spreading in association with materials and objects that move between buildings, cities, countries, and probably continents. Why it is sometimes found in large numbers is not yet clear. We saw no clear pattern in its spread from Hungary to other countries in Europe, and it has still not been recorded in some European countries, but this might also be related to false identification. This makes it important to provide an identification key for the synanthropic species found in homes and museums in Europe, and to tabulate morphological characteristics. We assume that the species is still spreading and will be found more frequently in buildings and homes, similarly to Austria, where it is already quite common. Thus far, no damage to museum objects can be attributed to *Ctenolepisma calvum*, but this might be expected if their numbers increase dramatically in particular locations; hence, the ghost silverfish remains worthy of further study.

## Data Availability

Data can be obtained from the authors.

## Supplementary Materials

The following are available online at https://www.mdpi.com/article/10.3390/insects13090855/s1, Table S1: List of specimens used for the DNA barcoding and the Neighbour-Joining tree construction, Table S2: Records of the species *C. calvum* in different European countries, including also “negative” records (evaluation by experts of museum pests and Apterygota), Table S3: Identification key for the synanthropic species in Central Europe, Table S4: Taxonomic and morphological species characters for Central Europe reported species.

## Appendix A. List of Austrian Museums

21er Haus: Modern art museum and storage, Vienna
Belved O: Obere Belvedere storage and museum, Vienna
Belved R: Belvedere conservation studio, Vienna
Belved U: Lower Belvedere and Orangery museum, Vienna
Depot N: storage Landessammlung Niederösterreich, Lower Austria
Depot NT: storage Landessammlung Niederösterreich, Lower Austria
Dom: Dom museum, Vienna
HGM: Military History Museum, Vienna
HGM Obj 15: storage of the Military History Museum, Vienna
HGM Obj 3: storage of the Military History Museum, Vienna
Hofburg: Imperial Palace, Vienna
Stor 1: art and object storage, Lower Austria
Mus 1-5: five different art and object museums with storages, Vienna
Klosterneuburg B: historic library Monastery Klosterneuburg, Lower Austria
Klosterneuburg A: archive of the Klosterneuburg Monastery, Lower Austria
Kunsttrans; commercial art storage, Vienna
Franzensburg: historic castle in Laxenburg, Lower Austria
Leopold: museum and storage, Vienna
Liechtenstein G: office of the museum, Vienna
Liechtenstein S: Stadtpalais Liechtenstein, storage and museum, Vienna
MAK: Museum for Applied Arts, Vienna
MAK Schlößl: history museum, Vienna
MAK Turm: second world war tower, storage, Vienna
MUMOK: Museum of Modern Art, Vienna
MUMOK H: Hafen storage, Vienna
NHM: Natural History Museum, museum and storage, Vienna
Niedersulz: Open art museum, storage, Niedersulz, Lower Austria
Salzburg D: Depot, storage, Salzburg
Salzburg K: Klessheim—Storage below Football stadium, Salzburg
Salzburg M: Monatsschlößl—17th-century hunting lodge located at Hellbrunn Palace
Salzburg R: neue Residenz—art, history and culture former Carolino-Augusteum museum,
Salzburg S: Spielzeug—Toy Museum, storage, Salzburg
Schönbrunn: Schönbrunn Palace, museum and storage, Vienna
TMW Breit: Technisches Museum Wien, storage, Vienna
TMW Har: Technisches Museum Wien, storage, Lower Austria
TMW: Technisches Museum Wien, Technical Museum; Vienna
WM Him: Wien Museum Storage in Himberg; Lower Austria

## Appendix B.

**Description of the record in Poland**

 **Author of observation:** Michal Grabowski
 **Observation date:** since 2014 until now—viable population in a sanitary installation of an apartment complex built in 2012
 **Coordinates:** 51.73644705121365, 19.471731822502928
 **Locality:** Łódź (Lodz), Poland

**Observation from iNaturalist:**

 **Author of observation:** Monika Miturová
 **Observation date:** 1 November 2021 09:13 CET
 **Submitted to iNaturalist:** 1 November 2021 09:21 CET
 **Coordinates:** 50.458141, 16.387447
 **Accuracy:** 4.86 km
 **Locality:** Szczytna, Poland
 **Link:** https://www.inaturalist.org/observations/99959118, accessed on 1 July 2022 [43]
