# Supplementary material for: Identification and Spread of the Ghost Silverfish (Ctenolepisma calvum) among Museums and Homes in Europe
Source: Insects. 2022 Sep 19;13(9):855. doi: 10.3390/insects13090855 (PMC9505982; doi:10.3390/insects13090855)
Supplement: Supplementary file 1 [file insects-13-00855-s001.zip › Appendix S1.pdf]

insects MDPI

Appendix S1 Description of the record in Poland

Author of observation: Michal Grabowski
Observation date: since 2014 until now – viable population in a sanitary installation of an apartment complex built in 2012
Coordinates: 51.73644705121365, 19.471731822502928
Locality: Łódź (Lodz), Poland

Observation from iNaturalist:
Author of observation: Monika Miturová
Observation date: 1 Nov 2021 · 09:13 CET
Submitted to iNaturalist: 1 Nov 2021 · 09:21 CET
Coordinates: 50.458141, 16.387447
Accuracy: 4.86km
Locality: Szczytna, Poland
Link: https://www.inaturalist.org/observations/99959118 [43]
