# Supplementary material for: Identification and Spread of the Ghost Silverfish (Ctenolepisma calvum) among Museums and Homes in Europe
Source: Insects. 2022 Sep 19;13(9):855. doi: 10.3390/insects13090855 (PMC9505982; doi:10.3390/insects13090855)
Supplement: Supplementary file 1 [file insects-13-00855-s001.zip › Table S1.pdf]

Table S1. List of specimens used for the DNA barcoding and the Neighbour-Joining tree construction.

**Species - IDs from BOLD**	**Project Code**	**Process ID**	**Sample ID**	**BIN**	**GenBank accession number**	**Locality**	**Lat**	**Lon**	**Sequence origin**
Ctenolepisma longicaudatum	ZYI	ZYI065-18	gbs001836	BOLD:AEE2038	-	Hobart, Tasmania, Australia	-42.5872	147.306	Graeme et al. 2019
Zygentoma	GBZYG	GBEPT734-14	GBOL04158	BOLD:ACA5333	-	Chemnitz, Germany	50.834	12.926	BOLD
Zygentoma	GBZYG	GBEPT732-14	GBOL04156	BOLD:ACA5333	-	Chemnitz, Germany	50.834	12.926	BOLD
Zygentoma	GBZYG	GBEPT729-14	GBOL04153	BOLD:ACA5333	-	Chemnitz, Germany	50.834	12.926	BOLD
Zygentoma	GBZYG	GBEPT728-14	GBOL04152	BOLD:ACA5333	-	Chemnitz, Germany	50.834	12.926	BOLD
Lepisma saccharinum	PLLEP	PLLEP011-21	Ajaz_Lepisma_2	BOLD:ACB9678	OP028709	Kalonka, Poland	51.8285	19.574	This study
Lepisma saccharinum	PLLEP	PLLEP010-21	AJaz_Lepisma_1	BOLD:ACB9678	OP028707	Kalonka, Poland	51.8285	19.574	This study

Lepisma saccharinum	PLLEP	PLLEP005-21	KBS_Lepisma_2	BOLD:ACB9678	OP028706	Lodz, Poland	51.7745	19.4766	This study
Lepisma saccharinum	PLLEP	PLLEP004-21	TR_Lepisma_2	BOLD:ACB9678	OP028705	Lodz, Poland	51.7767	19.5305	This study
Ctenolepisma calvum	PLLEP	PLLEP009-21	MG_Ctenolepisma_3	BOLD:ACA5333	OP028701	Lodz, Poland	51.7372	19.4721	This study
Ctenolepisma calvum	PLLEP	PLLEP008-21	MG_Ctenolepisma_2	BOLD:ACA5333	OP028703	Lodz, Poland	51.7372	19.4721	This study
Ctenolepisma calvum	PLLEP	PLLEP007-21	MG_Ctenolepisma_1	BOLD:ACA5333	OP028702	Lodz, Poland	51.7372	19.4721	This study
Lepisma saccharinum	PLLEP	PLLEP006-21	MG_mom_Lepisma_1	BOLD:ACB9678	OP028708	Lodz, Poland	51.7359	19.4716	This study
Lepisma saccharinum	PLLEP	PLLEP001-21	Ajab_Lepisma_1	BOLD:ACB9678	OP028710	Lodz, Poland	51.8014	19.4706	This study
Lepisma saccharinum	PLLEP	PLLEP003-21	KBS_Lepisma_1	BOLD:ACB9678	OP028704	Lodz, Poland	51.7745	19.4766	This study
Lepisma saccharinum	PLLEP	PLLEP002-21	TR_Lepisma_1	BOLD:ACB9678	OP028711	Lodz, Poland	51.7767	19.5305	This study
