# Supplementary material for: Identification and Spread of the Ghost Silverfish (Ctenolepisma calvum) among Museums and Homes in Europe
Source: Insects. 2022 Sep 19;13(9):855. doi: 10.3390/insects13090855 (PMC9505982; doi:10.3390/insects13090855)
Supplement: Supplementary file 1 [file insects-13-00855-s001.zip › Table S2.pdf]

Table S2 Records of the species *C. calvum* in different European countries, including also “negative” records (evaluation by experts of museum pests and Apterygota):

Country	Year of first record	Record / citation
Hungary	2003	Gábor Hegyessy 2021, published online https://www.izeltlabuak.hu/faj/szellemhalacska
Germany	2007	Data by S. Erlacher 2017, published online www.chemnitz.de
Austria	2010	Data by S. Erlacher, unpublished<br>2014 in a museum: Querner et al. 2017
Tenerife (Spain)	2012	inaturalist
Poland	2014	First record in this paper Querner et al. 2022<br>inaturalist
Finland	2017	Data by S. Erlacher, unpublished<br>inaturalist
Switzerland	2017	Data by S. Erlacher, unpublished<br>2019 in a museum: P. Querner, unpublished
Norway	2018	Published: Aak et al. 2021<br>Also in data by S. Erlacher, unpublished
Italy	2020	Data by S. Erlacher, unpublished
Russia	2020	inaturalist
Luxembourg	2021	Data by S. Erlacher, unpublished
Croatia	2021	inaturalist
Czech Republic	2021	Kulma et al. 2022
Spain	2021	inaturalist
Kosovo	2021	inaturalist

Slovenia	2021	inaturalist
Ukraine	2021	inaturalist
Portugal	2021	inaturalist
Slovakia	2022	inaturalist
Sweden	-	Niklas Apelqvist (oral communication, 2022)
Liechtenstein	-	P. Querner, not found in museums<br>Peter Niederklopfer (oral communication, 2022)
Netherlands	-	Matty Berg (oral communication, 2022)
France	-	Cyrille D' Haese (oral communication, 2022)
Belgium	-	Koen Lock (oral communication, 2022)
UK	-	David Pinniger (oral communication, 2022)
Denmark	-	Anne-Kathrine Kjerulff (oral communication, 2022)
