# Supplementary material for: Identification and Spread of the Ghost Silverfish (Ctenolepisma calvum) among Museums and Homes in Europe
Source: Insects. 2022 Sep 19;13(9):855. doi: 10.3390/insects13090855 (PMC9505982; doi:10.3390/insects13090855)
Supplement: Supplementary file 1 [file insects-13-00855-s001.zip › Table S3.pdf]

Table S3 Identification key for the synanthropic species in Central Europe:

1. Animals without eyes. 4–6 mm. In ant nests, rarely found in buildings.
Atelura fomicaria

or: Animal with eyes. Often found in buildings. **-> 2**

2. Scales on the abdomen are uniformly coloured. One or two pairs of abdominal stylets. **-> 3**

or: Abdomen with mixed dark and light-coloured scales. Three pairs of abdominal stylets. **-> 5**

3. 10th Tergit of the abdomen clearly longer than wide. Cerci (the two long appendices on the side) clearly shorter than the body, in living animals usually pointing diagonally backwards.
- Up to 11 mm.
- In buildings, sometimes also outside. *Lepisma saccharinum*

or: 10th Tergit of the abdomen clearly shorter than wide. Cerci shorter or as long as the body of the animal, in living animals usually held perpendicular (ca. 90°) to body axis. **-> 4**

4. Abdomen with hairs. Tergite of the thorax at the end with one pair of bristle combs. Cerci as long as the body. Scales grey (dark or light). Two pairs of abdominal stylets
- Up to 15 mm.
- In buildings, adults are also found in dry environments. Nymphs need humid environments *Ctenolepisma longicaudatum*

or: Abdomen without hairs. Tergite of the thorax at the end with 1 pair of individual bristles. Cerci ca. 2/3 of the body length. Scales white (to yellowish). One pair of abdominal stylets
- Ca. 8 mm.
- In buildings, in warm rooms. *Ctenolepisma calvum*

5. Abdomen with clear horizontal stripes.
- Up to 10 mm.
- In buildings, in hot rooms. *Thermobia domestica*

or: Abdomen with clear longitudinal stripes.
- 10-12 mm.
- In buildings, also outside, or the outside wall of buildings, often close to wood or under bark. *Ctenolepisma lineatum*

For further taxonomic and morphological characters see the supplementary Table 4.
