# Supplementary material for: Identification and Spread of the Ghost Silverfish (Ctenolepisma calvum) among Museums and Homes in Europe
Source: Insects. 2022 Sep 19;13(9):855. doi: 10.3390/insects13090855 (PMC9505982; doi:10.3390/insects13090855)
Supplement: Supplementary file 1 [file insects-13-00855-s001.zip › Table S4.pdf]

Table S4 Taxonomic and morphological species characters for Central Europe reported species:

Name	Shape of urotergite X	combs on urotergites	Combs on urosternites	Number of styli	Colour of scales	Tufts of feathered bristles at frontal part of head	Leg pigmentation	Length of abdominal appendeages	Ovipositor
Lepisma saccharinum	Lengthy (longer than wide)	No combs	No combs	2	at dorsal side uniformly grey, ventral side light	absent	absent	cerci/ body: <0.5<br>Cerci point obliquely backwards<br><br>terminal filament/ body: <0.5	
Thermobia domestica	broad subtriangular	**I:** 1+1<br>**II:** 2+2<br>**III:** 2+2<br>**IV:** 2+2<br>**V:** 2+2<br>**VI:** 2+2<br>**VII:** 2+2<br>**VIII:** 2+2<br>**IX:** 0<br>**X:** 1+1	**I:** 0<br>**II:** 0<br>**III:** 1<br>**IV:** 1+1+1<br>**V:** 1+1+1<br>**VI:** 1+1+1<br>**VII:** 1+1<br>**VIII:** 1+1	3(f) 3(m)	dark and light grey mixed with brown (body patterned in horizontal stripes of different scale colours)	present	nearly absent to absent (tibiae distally with slightly pigmented ring, tarsus 1 likewise with slightly pigmented ring)	cerci/ body:<br>(f) 0.8<br>(m) 0.9<br><br>terminal filament/ body:<br>(f) 1.0<br>(m) 0.8	Slender, 1-2 times length of styli IX
Ctenolepisma calvum	trapezoidal	**I:** 1+1<br>**II:** 3+3<br>**III:** 3+3<br>**IV:** 3+3<br>**V:** 3+3<br>**VI:** 2+2<br>**VII:** 2+2<br>**VIII:** 2+2<br>**IX:** 0<br>**X:** 1+1	**I:** 0<br>**II:** 0<br>**III:** 1+1<br>**IV:** 1+1<br>**V:** 1+1<br>**VI:** 1+1<br>**VII:** 1+1<br>**VIII:** 1+1	1(f) 1(m)	at dorsal side whitish to yellowish	present	absent	cerci/ body:<br>~0.66<br><br>terminal filament/ body:<br>~1.0	slightly surpasses styli IX
Ctenolepisma lineatum	broad subtriangular	**I:** 1+1<br>**II:** 3+3<br>**III:** 3+3<br>**IV:** 3+3<br>**V:** 3+3<br>**VI:** 3+3<br>**VII:** 3+3<br>**VIII:** 2+2<br>**IX:** 0<br>**X:** 1+1	**I:** 0<br>**II:** 0<br>**III:** 1+1<br>**IV:** 1+1<br>**V:** 1+1<br>**VI:** 1+1<br>**VII:** 1+1<br>**VIII:** 1+1	3(f) 3(m)	at dorsal side greyish and light brown (the light brownish scales form longitudina stripes)	present	present moderately to strong	cerci/ body: ~1.0<br><br>terminal filament/ body: ~1.0	long, with 47–52 divisions, 0.9–2 times length of styli IX

Ctenolepisma longicaudatum	trapezoidal	**I:** 1+1<br>**II:** 3+3<br>**III:** 3+3<br>**IV:** 3+3<br>**V:** 3+3<br>**VI:** 3+3<br>**VII:** 2+2<br>**VIII:** 2+2<br>**IX:** 0<br>**X:** 1+1	**I:** 0<br>**II:** 0<br>**III:** 1+1<br>**IV:** 1+1<br>**V:** 1+1<br>**VI:** 1+1<br>**VII:** 1+1<br>**VIII:** 1+1	2(f) 2(m)	at dorsal side uniformly grey, seldom silverish to whitish	present	absent	cerci/ body: ~0.8<br><br>terminal filament/ body: ~1.0	long, 2 times length of styli IX
Ctenolepisma targionium	trapezoidal	**I:** 2+2<br>**II:** 3+3<br>**III:** 3+3<br>**IV:** 3+3<br>**V:** 3+3<br>**VI:** 2+2<br>**VII:** 2+2<br>**VIII:** 1+1<br>**IX:** 0<br>**X:** 1+1		2(f) 1(m)	at dorsal side uniformly light grey, at ventral side whitish	present	absent (yellowish white to white)	cerci/ body: ~0.66<br><br>terminal filament/ body: ~0.66	
Ctenolepisma ciliatum	trapezoidal	**I:** 1+1<br>**II:** 3+3<br>**III:** 3+3<br>**IV:** 3+3<br>**V:** 3+3<br>**VI:** 3+3<br>**VII:** 2+2<br>**VIII:** 2+2<br>**IX:** 0<br>**X:** 1+1	**I:** 0<br>**II:** 0<br>**III:** 1+1<br>**IV:** 1+1<br>**V:** 1+1<br>**VI:** 1+1<br>**VII:** 1+1<br>**VIII:** 1+1	2(f) 2(m)	on dorsal side uniformly brown to greyish brown; ventral side lighter	present	epidermic pigment more or less dark, but always distinct, usually very intense on the legs	terminal filament shorter than body length, cerci shorter than filament	short to relatively long,<br>1–1.5 times length of styli IX
Ctenolepisma rothschildi	trapezoidal	**I:** 1+1<br>**II:** 3+3<br>**III:** 3+3<br>**IV:** 3+3<br>**V:** 3+3<br>**VI:** 2+2<br>**VII:** 2+2<br>**VIII:** 2+2<br>**IX:** 0<br>**X:** 1+1	**I:** 0<br>**II:** 0<br>**III:** 1+1<br>**IV:** 1+1<br>**V:** 1+1<br>**VI:** 1+1<br>**VII:** 1+1<br>**VIII:** 1+1	2(f) 2(m)	on dorsal side uniformly very dark; ventral side light grey/straw colour	present	without much pigment, but pigmented	cerci/ body: 0.6<br><br>terminal filament/ body: 0.6	thin, very long
